# (*E*)-1-(2-Thien­yl)-3-(3,4,5-trimethoxy­phen­yl)prop-2-en-1-one^1^

**DOI:** 10.1107/S1600536809021850

**Published:** 2009-06-13

**Authors:** Thitipone Suwunwong, Suchada Chantrapromma, Paradorn Pakdeevanich, Hoong-Kun Fun

**Affiliations:** a Crystal Materials Research Unit, Department of Chemistry, Faculty of Science, Prince of Songkla University, Hat-Yai, Songkhla 90112, Thailand; b Department of Physics, Faculty of Science, Prince of Songkla University, Hat-Yai, Songkhla 90112, Thailand; c X-ray Crystallography Unit, School of Physics, Universiti Sains Malaysia, 11800 USM, Penang, Malaysia

## Abstract

The mol­ecule of the title heteroaryl chalcone, C_16_H_16_O_4_S, which consists of substituted thio­phene and benzene rings bridged by the prop-2-en-1-one group, is slightly twisted. The dihedral angle between the thio­phene and 3,4,5-trimethoxy­phenyl rings is 12.18 (4)°. The three meth­oxy groups have two different conformations; two meth­oxy groups are coplanar [C—O—C—C torsion angles = −1.38 (12) and 0.47 (12)°] whereas the third is (-)-synclinal with the benzene ring. In the crystal structure, adjacent mol­ecules are linked in a face-to-side manner into chains along the *c* axis by weak C—H⋯O(enone) inter­actions. These chains are stacked along the *b* axis by weak C—H⋯O(meth­oxy) inter­actions.

## Related literature

For bond-length data, see: Allen *et al.* (1987 ▶). For hydrogen-bond motifs, see: Bernstein *et al.* (1995 ▶). For related structures, see: Chantrapromma *et al.* (2009 ▶); Patil *et al.* (2006 ▶; 2007 ▶); Suwunwong *et al.* (2009*a* ▶,*b* ▶). For background to and applications of chalcones, see: Dimmock *et al.* (1999 ▶); Go *et al.* (2005 ▶); Jung *et al.* (2008 ▶); Ni *et al.* (2004 ▶); Patil *et al.* (2007 ▶); Patil & Dharmaprakash (2008 ▶). For the stability of the temperature controller used in the data collection, see: Cosier & Glazer, (1986 ▶).
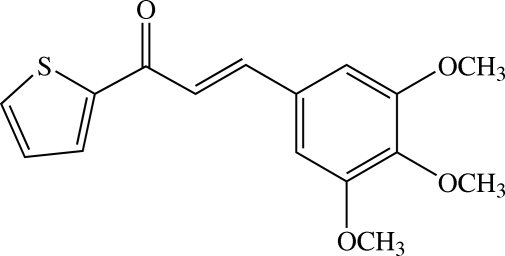


## Experimental

### 

#### Crystal data

C_16_H_16_O_4_S
*M* *_r_* = 304.36
Orthorhombic, 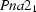

*a* = 25.3323 (8) Å
*b* = 3.9816 (1) Å
*c* = 14.0163 (4) Å
*V* = 1413.73 (7) Å^3^
*Z* = 4
Mo *K*α radiation
μ = 0.24 mm^−1^
*T* = 100 K
0.58 × 0.31 × 0.21 mm

#### Data collection

Bruker APEXII CCD area-detector diffractometer
Absorption correction: multi-scan (*SADABS*; Bruker, 2005 ▶) *T*_min_ = 0.872, *T*_max_ = 0.951
56940 measured reflections
7416 independent reflections
7177 reflections with *I* > 2σ(*I*)
*R*_int_ = 0.028

#### Refinement

*R*[*F* ^2^ > 2σ(*F* ^2^)] = 0.033
*wR*(*F* ^2^) = 0.099
*S* = 1.10
7416 reflections
193 parameters
1 restraint
H-atom parameters constrained
Δρ_max_ = 0.66 e Å^−3^
Δρ_min_ = −0.54 e Å^−3^
Absolute structure: Flack (1983 ▶), 3588 Friedel pairs
Flack parameter: 0.04 (4)

### 

Data collection: *APEX2* (Bruker, 2005 ▶); cell refinement: *SAINT* (Bruker, 2005 ▶); data reduction: *SAINT*; program(s) used to solve structure: *SHELXTL* (Sheldrick, 2008 ▶); program(s) used to refine structure: *SHELXTL*; molecular graphics: *SHELXTL*; software used to prepare material for publication: *SHELXTL* and *PLATON* (Spek, 2009 ▶).

## Supplementary Material

Crystal structure: contains datablocks global, I. DOI: 10.1107/S1600536809021850/sj2627sup1.cif

Structure factors: contains datablocks I. DOI: 10.1107/S1600536809021850/sj2627Isup2.hkl

Additional supplementary materials:  crystallographic information; 3D view; checkCIF report

## Figures and Tables

**Table 1:** Hydrogen-bond geometry (Å, °)

*D*—H⋯*A*	*D*—H	H⋯*A*	*D*⋯*A*	*D*—H⋯*A*
C1—H1*A*⋯O1^i^	0.93	2.52	3.1827 (14)	129
C15—H15*C*⋯O3^ii^	0.96	2.39	3.3340 (11)	169

Symmetry codes: (i) 
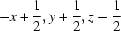
; (ii) 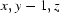.

**Fig. 1.**
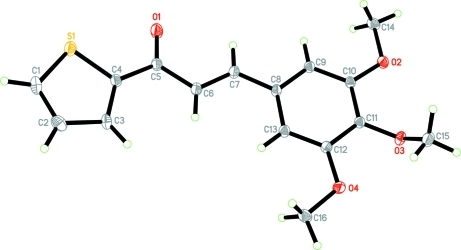
The molecular structure of the title compound, showing 50% probability displacement ellipsoids and the atom-numbering scheme.

**Fig. 2.**
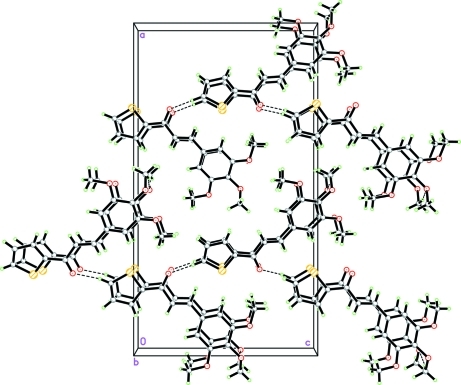
The crystal packing of the title compound viewed along the b axis, showing chains running along the c axis. Weak C—H···O interactions are shown as dashed lines.

**Fig. 3.**
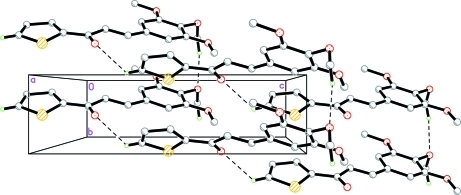
The crystal packing of the title compound viewed along the a axis, showing chains stacking along the b axis. Weak C—H···O interactions are shown as dashed lines and hydrogen atoms not involved in C—H···O interactions were omitted for clarity.

## supplementary crystallographic information

### Comment

Chalcone or 1,3-diaryl-2-propen-1-one, originally isolated from natural sources, and its derivatives are known to display a variety of biological activities, demonstrating analgesic, anti-inflammatory, antibacterial and antimycotic properties (Dimmock *et al.*, 1999; Go *et al.*, 2005; Ni *et al.*, 2004).  Moreover synthetic chalcones have also been found to be non-linear optical (NLO) (Patil & Dharmaprakash, 2008) and electro-active fluorescent materials (Jung *et al.*, 2008). We have previously reported the synthesis and crystal structures of chalcone derivatives (Chantrapromma *et al.*, 2009; Suwunwong *et al.*, 2009*a*, *b*). Our research into the  NLO and biological properties of chalcone derivatives led us to synthesize the title heteroaryl chalcone (I). (I) crystallizes in the non-centrosymmetric orthorhombic space group *Pna*2_1_ and should therefore exhibit second-order nonlinear optical properties.

The molecule of the title heteroaryl chalcone (Fig. 1) exists in an *E* configuration with respect to the C6═C7 double bond [1.3437 (11) Å] with a C5–C6–C7–C8 torsion angle 176.81 (8)°. The whole molecule is twisted as shown by the interplanar angle between thiophene and 3,4,5-trimethoxyphenyl rings being 12.18 (4)°. The propenone unit (C5—C7/O1) is also twisted with the O1–C5–C6–C7 torsion angle 10.94 (15)°. The three substituted methoxy groups of 3,4,5-trimethoxyphenyl unit have two different orientations: two methoxy groups (at the C10 and C12 positions) are co-planar with the phenyl ring with torsion angles C14–O2–C10–C9 = -1.38 (12)° and C16–O4–C12–C13 = 0.47 (12)° whereas the one at C11 is (-)-*syn*-clinally attached with the C15–O3–C11–C12 torsion angle  -76.76 (10)°. In the structure, weak intramolecular C7—H7A···O1 and C15—H15B···O4  interactions generate S(5) and S(6) ring motifs, respectively (Bernstein *et al.*, 1995). The bond distances have normal values (Allen *et al.*, 1987) and bond lengths and angles are comparable with closely related structures (Chantrapromma *et al.*, 2009; Patil *et al.*, 2006; 2007; Suwunwong *et al.*, 2009*a*, *b*).

In the crystal packing, the adjacent molecules are linked in a face-to-side manner into chains along the *c* axis through the enone unit by weak C1—H1A···O1 interactions (Fig. 2, Table 1). Weak C15—H15C···O3 interactions involving one of methoxy groups further stack these chains along the *b* axis (Fig. 3, Table 1).

### Experimental

The title compound was synthesized by the condensation of 3,4,5-trimethoxybenzaldehyde (0.40 g, 2 mmol) with 2-acetylthiophene (0.35 ml, 2 mmol) in ethanol (30 ml) in the presence of 30% NaOH (aq) (5 ml). After stirring for 3 h in ice bath at 278 K, the resulting pale yellow solid was collected by filtration, washed with distilled water, dried in air and purified by repeated recrystallization from acetone (72% yield). Pale yellow block-shaped single crystals of the title compound suitable for *x*-ray structure determination were recrystalized from acetone/ethanol (1:1 *v*/*v*) by the slow evaporation of the solvent at room temperature after several days, Mp. 420–421 K.

### Refinement

All H atoms were placed in calculated positions, with C—H = 0.93 Å, *U*_iso_ = 1.2*U*_eq_(C) for aromatic and CH and C—H = 0.96 Å, *U*_iso_ = 1.5*U*_eq_(C) for CH_3_ atoms. A rotating group model was used for the methyl groups. The highest residual electron density peak is located at 0.22 Å from C3 and the deepest hole is located at 0.20 Å from S1.

### Crystal data

**Table:**

C_16_H_16_O_4_S	*D*_x_ = 1.430 Mg m^−^^3^
*M**_r_* = 304.36	Melting point = 420–421 K
Orthorhombic, *P**n**a*2_1_	Mo *K*α radiation, λ = 0.71073 Å
Hall symbol: P 2c -2n	Cell parameters from 7416 reflections
*a* = 25.3323 (8) Å	θ = 2.2–37.5°
*b* = 3.9816 (1) Å	µ = 0.24 mm^−^^1^
*c* = 14.0163 (4) Å	*T* = 100 K
*V* = 1413.73 (7) Å^3^	Block, pale yellow
*Z* = 4	0.58 × 0.31 × 0.21 mm
*F*(000) = 640	

### Data collection

**Table:**

Bruker APEXII CCD area-detector diffractometer	7416 independent reflections
Radiation source: sealed tube	7177 reflections with *I* > 2σ(*I*)
graphite	*R*_int_ = 0.028
φ and ω scans	θ_max_ = 37.5°, θ_min_ = 2.2°
Absorption correction: multi-scan (*SADABS*; Bruker, 2005)	*h* = −43→43
*T*_min_ = 0.872, *T*_max_ = 0.951	*k* = −6→6
56940 measured reflections	*l* = −23→24

### Refinement

**Table:**

Refinement on *F*^2^	Secondary atom site location: difference Fourier map
Least-squares matrix: full	Hydrogen site location: inferred from neighbouring sites
*R*[*F*^2^ > 2σ(*F*^2^)] = 0.033	H-atom parameters constrained
*wR*(*F*^2^) = 0.099	*w* = 1/[σ^2^(*F*_o_^2^) + (0.0672*P*)^2^ + 0.1429*P*] where *P* = (*F*_o_^2^ + 2*F*_c_^2^)/3
*S* = 1.10	(Δ/σ)_max_ = 0.001
7416 reflections	Δρ_max_ = 0.66 e Å^−^^3^
193 parameters	Δρ_min_ = −0.54 e Å^−^^3^
1 restraint	Absolute structure: Flack (1983), 3588 Friedel pairs
Primary atom site location: structure-invariant direct methods	Flack parameter: 0.04 (4)

### Special details

**Table:**

Experimental. The crystal was placed in the cold stream of an Oxford Cryosystems Cobra open-flow nitrogen cryostat (Cosier & Glazer, 1986) operating at 120.0 (1) K.
Geometry. All e.s.d.'s (except the e.s.d. in the dihedral angle between two l.s. planes) are estimated using the full covariance matrix. The cell e.s.d.'s are taken into account individually in the estimation of e.s.d.'s in distances, angles and torsion angles; correlations between e.s.d.'s in cell parameters are only used when they are defined by crystal symmetry. An approximate (isotropic) treatment of cell e.s.d.'s is used for estimating e.s.d.'s involving l.s. planes.
Refinement. Refinement of *F*^2^ against ALL reflections. The weighted *R*-factor *wR* and goodness of fit *S* are based on *F*^2^, conventional *R*-factors *R* are based on *F*, with *F* set to zero for negative *F*^2^. The threshold expression of *F*^2^ > σ(*F*^2^) is used only for calculating *R*-factors(gt) *etc*. and is not relevant to the choice of reflections for refinement. *R*-factors based on *F*^2^ are statistically about twice as large as those based on *F*, and *R*- factors based on ALL data will be even larger.

### Fractional atomic coordinates and isotropic or equivalent isotropic displacement parameters (Å^2^)

**Table:**

	*x*	*y*	*z*	*U*_iso_*/*U*_eq_	
S1	0.249987 (8)	0.46794 (6)	0.47989 (2)	0.01892 (5)	
O1	0.25370 (3)	0.4617 (3)	0.68801 (7)	0.02618 (18)	
O2	0.40965 (3)	0.51095 (18)	1.13056 (5)	0.01662 (11)	
O3	0.49824 (2)	0.83176 (19)	1.08244 (5)	0.01615 (10)	
O4	0.51691 (3)	1.02802 (19)	0.90201 (5)	0.01753 (11)	
C1	0.28105 (4)	0.5872 (3)	0.37853 (6)	0.02121 (16)	
H1A	0.2679	0.5465	0.3177	0.025*	
C2	0.32771 (4)	0.7503 (3)	0.39595 (7)	0.02174 (16)	
H2A	0.3493	0.8331	0.3477	0.026*	
C3	0.34021 (3)	0.7815 (2)	0.49530 (5)	0.01517 (12)	
H3A	0.3702	0.8844	0.5200	0.018*	
C4	0.29890 (3)	0.6283 (2)	0.55061 (6)	0.01440 (12)	
C5	0.29401 (3)	0.5880 (2)	0.65390 (6)	0.01617 (13)	
C6	0.33914 (3)	0.6919 (2)	0.71401 (6)	0.01613 (13)	
H6A	0.3662	0.8202	0.6878	0.019*	
C7	0.34122 (3)	0.6018 (2)	0.80632 (6)	0.01549 (13)	
H7A	0.3122	0.4836	0.8294	0.019*	
C8	0.38341 (3)	0.6666 (2)	0.87483 (5)	0.01329 (11)	
C9	0.37539 (3)	0.5559 (2)	0.96864 (6)	0.01372 (11)	
H9A	0.3441	0.4479	0.9849	0.016*	
C10	0.41415 (3)	0.6073 (2)	1.03751 (5)	0.01261 (11)	
C11	0.46146 (3)	0.7683 (2)	1.01284 (5)	0.01296 (11)	
C12	0.46951 (3)	0.8767 (2)	0.91840 (5)	0.01333 (12)	
C13	0.43078 (3)	0.8266 (2)	0.84955 (6)	0.01399 (12)	
H13A	0.4362	0.8985	0.7872	0.017*	
C14	0.36101 (4)	0.3538 (2)	1.15750 (6)	0.01816 (14)	
H14A	0.3619	0.3001	1.2243	0.027*	
H14B	0.3563	0.1516	1.1212	0.027*	
H14C	0.3322	0.5043	1.1452	0.027*	
C15	0.54355 (3)	0.6178 (2)	1.07845 (8)	0.02027 (15)	
H15A	0.5681	0.6817	1.1274	0.030*	
H15B	0.5601	0.6391	1.0172	0.030*	
H15C	0.5329	0.3890	1.0881	0.030*	
C16	0.52661 (4)	1.1439 (3)	0.80695 (6)	0.01969 (15)	
H16A	0.5598	1.2597	0.8049	0.030*	
H16B	0.4989	1.2941	0.7880	0.030*	
H16C	0.5276	0.9555	0.7642	0.030*	

### Atomic displacement parameters (Å^2^)

**Table:**

	*U*^11^	*U*^22^	*U*^33^	*U*^12^	*U*^13^	*U*^23^
S1	0.01673 (9)	0.02390 (10)	0.01613 (9)	−0.00032 (6)	−0.00311 (6)	0.00034 (9)
O1	0.0181 (3)	0.0441 (5)	0.0164 (3)	−0.0106 (3)	−0.0019 (2)	0.0083 (3)
O2	0.0169 (2)	0.0225 (3)	0.0104 (2)	−0.0028 (2)	0.00034 (19)	0.00240 (19)
O3	0.0152 (2)	0.0203 (3)	0.0130 (2)	−0.00066 (19)	−0.00317 (18)	−0.0026 (2)
O4	0.0148 (2)	0.0241 (3)	0.0136 (2)	−0.0053 (2)	0.0005 (2)	0.0021 (2)
C1	0.0243 (4)	0.0266 (4)	0.0127 (3)	0.0053 (3)	−0.0033 (3)	−0.0005 (3)
C2	0.0220 (4)	0.0247 (4)	0.0185 (3)	0.0027 (3)	0.0059 (3)	0.0038 (3)
C3	0.0144 (3)	0.0203 (3)	0.0108 (3)	0.0051 (2)	−0.0017 (2)	−0.0003 (2)
C4	0.0135 (3)	0.0168 (3)	0.0129 (3)	0.0002 (2)	−0.0012 (2)	0.0020 (2)
C5	0.0142 (3)	0.0215 (3)	0.0128 (3)	−0.0013 (3)	−0.0015 (2)	0.0025 (2)
C6	0.0147 (3)	0.0199 (3)	0.0138 (3)	−0.0014 (2)	−0.0017 (2)	0.0012 (2)
C7	0.0134 (3)	0.0206 (3)	0.0125 (3)	−0.0006 (2)	−0.0014 (2)	0.0006 (2)
C8	0.0123 (3)	0.0168 (3)	0.0108 (2)	0.0004 (2)	−0.0006 (2)	−0.0002 (2)
C9	0.0125 (3)	0.0169 (3)	0.0117 (3)	−0.0006 (2)	−0.0001 (2)	0.0002 (2)
C10	0.0128 (3)	0.0149 (3)	0.0101 (3)	0.0006 (2)	0.0003 (2)	0.0001 (2)
C11	0.0130 (3)	0.0153 (3)	0.0105 (2)	−0.0002 (2)	−0.0003 (2)	−0.0003 (2)
C12	0.0127 (3)	0.0157 (3)	0.0116 (3)	−0.0003 (2)	0.0010 (2)	−0.0007 (2)
C13	0.0129 (3)	0.0178 (3)	0.0113 (3)	0.0000 (2)	0.0002 (2)	0.0001 (2)
C14	0.0186 (3)	0.0206 (4)	0.0152 (3)	−0.0016 (3)	0.0039 (2)	0.0030 (3)
C15	0.0174 (3)	0.0188 (3)	0.0246 (4)	0.0001 (3)	−0.0066 (3)	−0.0001 (3)
C16	0.0171 (3)	0.0252 (4)	0.0168 (3)	−0.0013 (3)	0.0032 (3)	0.0051 (3)

### Geometric parameters (Å, °)

**Table:**

S1—C1	1.6921 (11)	C7—C8	1.4598 (11)
S1—C4	1.7104 (8)	C7—H7A	0.9300
O1—C5	1.2347 (11)	C8—C9	1.4015 (11)
O2—C10	1.3642 (10)	C8—C13	1.4040 (11)
O2—C14	1.4325 (11)	C9—C10	1.3920 (11)
O3—C11	1.3725 (10)	C9—H9A	0.9300
O3—C15	1.4304 (12)	C10—C11	1.4023 (11)
O4—C12	1.3630 (10)	C11—C12	1.4073 (11)
O4—C16	1.4312 (11)	C12—C13	1.3905 (11)
C1—C2	1.3704 (15)	C13—H13A	0.9300
C1—H1A	0.9300	C14—H14A	0.9600
C2—C3	1.4335 (13)	C14—H14B	0.9600
C2—H2A	0.9300	C14—H14C	0.9600
C3—C4	1.4382 (12)	C15—H15A	0.9600
C3—H3A	0.9300	C15—H15B	0.9600
C4—C5	1.4619 (12)	C15—H15C	0.9600
C5—C6	1.4792 (12)	C16—H16A	0.9600
C6—C7	1.3437 (11)	C16—H16B	0.9600
C6—H6A	0.9300	C16—H16C	0.9600
			
C1—S1—C4	92.57 (5)	O2—C10—C9	124.24 (7)
C10—O2—C14	116.54 (7)	O2—C10—C11	115.83 (7)
C11—O3—C15	114.04 (7)	C9—C10—C11	119.93 (7)
C12—O4—C16	116.78 (7)	O3—C11—C10	119.29 (7)
C2—C1—S1	112.61 (7)	O3—C11—C12	120.91 (7)
C2—C1—H1A	123.7	C10—C11—C12	119.73 (7)
S1—C1—H1A	123.7	O4—C12—C13	124.62 (7)
C1—C2—C3	113.88 (8)	O4—C12—C11	114.94 (7)
C1—C2—H2A	123.1	C13—C12—C11	120.43 (7)
C3—C2—H2A	123.1	C12—C13—C8	119.54 (7)
C2—C3—C4	109.02 (8)	C12—C13—H13A	120.2
C2—C3—H3A	125.5	C8—C13—H13A	120.2
C4—C3—H3A	125.5	O2—C14—H14A	109.5
C3—C4—C5	129.94 (7)	O2—C14—H14B	109.5
C3—C4—S1	111.91 (6)	H14A—C14—H14B	109.5
C5—C4—S1	118.14 (6)	O2—C14—H14C	109.5
O1—C5—C4	119.89 (8)	H14A—C14—H14C	109.5
O1—C5—C6	122.19 (8)	H14B—C14—H14C	109.5
C4—C5—C6	117.90 (7)	O3—C15—H15A	109.5
C7—C6—C5	120.26 (8)	O3—C15—H15B	109.5
C7—C6—H6A	119.9	H15A—C15—H15B	109.5
C5—C6—H6A	119.9	O3—C15—H15C	109.5
C6—C7—C8	127.94 (8)	H15A—C15—H15C	109.5
C6—C7—H7A	116.0	H15B—C15—H15C	109.5
C8—C7—H7A	116.0	O4—C16—H16A	109.5
C9—C8—C13	120.22 (7)	O4—C16—H16B	109.5
C9—C8—C7	117.10 (7)	H16A—C16—H16B	109.5
C13—C8—C7	122.68 (7)	O4—C16—H16C	109.5
C10—C9—C8	120.15 (7)	H16A—C16—H16C	109.5
C10—C9—H9A	119.9	H16B—C16—H16C	109.5
C8—C9—H9A	119.9		
			
C4—S1—C1—C2	−0.66 (8)	C14—O2—C10—C11	178.47 (7)
S1—C1—C2—C3	0.53 (11)	C8—C9—C10—O2	179.44 (8)
C1—C2—C3—C4	−0.06 (11)	C8—C9—C10—C11	−0.40 (12)
C2—C3—C4—C5	178.30 (9)	C15—O3—C11—C10	106.33 (9)
C2—C3—C4—S1	−0.43 (9)	C15—O3—C11—C12	−76.76 (10)
C1—S1—C4—C3	0.62 (7)	O2—C10—C11—O3	−2.98 (11)
C1—S1—C4—C5	−178.27 (7)	C9—C10—C11—O3	176.88 (8)
C3—C4—C5—O1	176.49 (10)	O2—C10—C11—C12	−179.93 (7)
S1—C4—C5—O1	−4.85 (13)	C9—C10—C11—C12	−0.07 (12)
C3—C4—C5—C6	−5.52 (14)	C16—O4—C12—C13	0.47 (12)
S1—C4—C5—C6	173.14 (7)	C16—O4—C12—C11	−179.67 (8)
O1—C5—C6—C7	10.94 (15)	O3—C11—C12—O4	3.51 (11)
C4—C5—C6—C7	−166.99 (8)	C10—C11—C12—O4	−179.59 (7)
C5—C6—C7—C8	176.81 (8)	O3—C11—C12—C13	−176.63 (8)
C6—C7—C8—C9	177.83 (9)	C10—C11—C12—C13	0.27 (12)
C6—C7—C8—C13	−3.34 (14)	O4—C12—C13—C8	179.86 (8)
C13—C8—C9—C10	0.69 (12)	C11—C12—C13—C8	0.01 (12)
C7—C8—C9—C10	179.55 (8)	C9—C8—C13—C12	−0.49 (12)
C14—O2—C10—C9	−1.38 (12)	C7—C8—C13—C12	−179.29 (8)

### Hydrogen-bond geometry (Å, °)

**Table:**

*D*—H···*A*	*D*—H	H···*A*	*D*···*A*	*D*—H···*A*
C1—H1A···O1^i^	0.93	2.52	3.1827 (14)	129
C7—H7A···O1	0.93	2.48	2.8243 (12)	102
C15—H15B···O4	0.96	2.49	3.0396 (13)	116
C15—H15C···O3^ii^	0.96	2.39	3.3340 (11)	169
C7—H7A···O1	0.93	2.48	2.8243 (12)	102
C15—H15B···O4	0.96	2.49	3.0396 (13)	116

Symmetry codes: (i) −*x*+1/2, *y*+1/2, *z*−1/2;  (ii) *x*, *y*−1, *z*.

## References

[bb1] Allen, F. H., Kennard, O., Watson, D. G., Brammer, L., Orpen, A. G. & Taylor, R. (1987). *J. Chem. Soc. Perkin Trans. 2*, pp. S1–19.

[bb2] Bernstein, J., Davis, R. E., Shimoni, L. & Chang, N.-L. (1995). *Angew. Chem. Int. Ed. Engl* **34**, 1555–1573.

[bb3] Bruker (2005). *APEX2*, *SAINT* and *SADABS* Bruker AXS Inc., Madison, Wisconsin, USA.

[bb4] Chantrapromma, S., Suwunwong, T., Karalai, C. & Fun, H.-K. (2009). *Acta Cryst.* E**65**, o893–o894. 10.1107/S1600536809010496 PMC2968789 21582601

[bb5] Cosier, J. & Glazer, A. M. (1986). *J. Appl. Cryst.* **19**, 105–107.

[bb6] Dimmock, J. R., Elias, D. W., Beazely, M. A. & Kandepu, N. M. (1999). *Curr. Med. Chem* **6**, 1125–1149. 10519918

[bb7] Flack, H. D. (1983). *Acta Cryst.* A**39**, 876–881.

[bb8] Go, M.-L., Wu, X. & Liu, X.-L. (2005). *Curr. Med. Chem* **12**, 483–499.

[bb9] Jung, Y. J., Son, K. I., Oh, Y. E. & Noh, D. Y. (2008). *Polyhedron*, **27**, 861–867.

[bb10] Ni, L., Meng, C. Q. & Sikorski, J. A. (2004). *Expert Opin. Ther. Patents*, **14**, 1669–1691.

[bb11] Patil, P. S., Chantrapromma, S., Fun, H.-K., Dharmaprakash, S. M. & Babu, H. B. R. (2007). *Acta Cryst* E**63**, o2612.

[bb12] Patil, P. S. & Dharmaprakash, S. M. (2008). *Mater. Lett* **62**, 451–453.

[bb13] Patil, P. S., Rosli, M. M., Fun, H.-K., Razak, I. A., Puranik, V. G. & Dharmaprakash, S. M. (2006). *Acta Cryst.* E**62**, o4798–o4799.

[bb14] Sheldrick, G. M. (2008). *Acta Cryst.* A**64**, 112–122. 10.1107/S0108767307043930 18156677

[bb15] Spek, A. L. (2009). *Acta Cryst.* D**65**, 148–155. 10.1107/S090744490804362X PMC2631630 19171970

[bb16] Suwunwong, T., Chantrapromma, S. & Fun, H.-K. (2009*a*). *Acta Cryst.* E**65**, o120. 10.1107/S1600536808041780 PMC2968041 21581582

[bb17] Suwunwong, T., Chantrapromma, S., Karalai, C., Pakdeevanich, P. & Fun, H.-K. (2009*b*). *Acta Cryst.* E**65**, o420–o421. 10.1107/S1600536809003122 PMC2968141 21582009

